# Compensation of Heat Effect in Dielectric Barrier Discharge (DBD) Plasma System for Radar Cross-Section (RCS) Reduction

**DOI:** 10.3390/s23167121

**Published:** 2023-08-11

**Authors:** Jinwoo Jung, Changseok Cho, Minsu Choi, Shinjae You, Jungje Ha, Hyunsoo Lee, Cheonyoung Kim, Ilyoung Oh, Yongshik Lee

**Affiliations:** 1 Department of Electrical and Electronic Engineering, Yonsei University, Seoul 03722, Republic of Korea; jjw1805@yonsei.ac.kr (J.J.); changseok.cho@yonsei.ac.kr (C.C.); 2 Department of Physics, Chungnam National University, Daejeon 34134, Republic of Korea; bss125576@naver.com (M.C.); sjyou@cnu.ac.kr (S.Y.); 3 Agency for Defense Development, Daejeon 34186, Republic of Korea; jungjeha@add.re.kr (J.H.); hslee83@add.re.kr (H.L.); cykim93@add.re.kr (C.K.); 4 Department of Information and Electronic Engineering, Dongyang Mirae University, Seoul 08221, Republic of Korea; pinokio13@dongyang.ac.kr

**Keywords:** dielectric barrier discharge (DBD), frequency selected surface (FSS), radar cross-section (RCS), plasma

## Abstract

In this study, the problems encountered in radar cross-section (RCS) measurement experiments utilizing a dielectric barrier discharge (DBD) plasma system are examined and an effective solution is proposed. A DBD plasma system generates heat due to the high bias voltage required for plasma generation. The thermal-induced structural deformation of the DBD structure caused by this high voltage and its impact on RCS measurements are analyzed. In addition, techniques for minimizing the thermal-induced deformation and compensation methods for addressing the minimized deformation are proposed. Furthermore, RCS measurements are conducted on two kinds of DBD structures using the proposed method to experimentally demonstrate the improved agreement between the simulation and measurement results. For both structures, the RCS experimental results are in very good agreement with the simulation results, which enables accurate plasma characterization. In conclusion, it can be expected that the proposed method can be used to provide more accurate RCS measurements on various DBD structures that generate high heat.

## 1. Introduction

Due to the direct impact on the survivability of weapon systems, the significance of low-observable techniques has increased in the modern era of highly advanced military technologies. Since World War II, significant attention has been given to low-observable technologies in the RF/microwave frequency bands, specifically aimed at reducing the radar cross-section (RCS) of various targets [1,2,3,4,5,6,7,8]. The generator can be strategically attached at the location that contributes the most to the RCS to enhance the survivability of the system.

The initial approach to RCS reduction involves the utilization of radar-absorbing materials (RAMs) or radar-absorbing structures (RASs), which absorb incoming radar signals and effectively decrease them [9,10,11,12,13]. However, these methods can be costly, not only in terms of manufacturing but also for maintenance purposes. A secondary technique relies on optimizing the shape of the target to minimize its electromagnetic size. For instance, the F-117 fighter aircraft has been specifically designed to have an RCS as low as that of a hummingbird [14]. Nevertheless, this technique may have limitations regarding certain incident angles and can potentially compromise the aerodynamic performance of the aircraft.

As another method for reducing the RCS, plasma-based technologies have recently attracted attention because of their ability to absorb and attenuate electromagnetic energy [15]. Compared with the aforementioned techniques, plasma methods are low-cost and have fewer restrictions in terms of size and weight, because the generator can simply be attached at the hot spots that contribute most effectively to its RCS. Due to the advantages of the plasma, it has been extensively investigated for reducing RCS with various plasma structures [16,17].

A previous study confirmed the stealth effect by applying an inductively coupled plasma generator to the S-shaped inlet of an aircraft and analyzing the reduction in the RCS through measurements [16]. It was observed that as the discharge power increased, the RCS reduction effect also increased. For an incident power level of 500 W, a reduction of up to 25 dB was achieved at 6 GHz. However, the use of an inductively coupled plasma generator necessitates a separate cooling device for stable operation due to the high temperatures generated during plasma generation. Moreover, there are limitations to increasing the plasma generation area, resulting in increased volume and weight, making it unsuitable for aircraft applications.

The dielectric barrier discharge (DBD) plasma generator, on the other hand, is relatively thin and generates less heat during plasma generation compared to inductively coupled plasma (ICP) [18,19,20,21]. However, the DBD plasma generator structure also requires the application of a high bias voltage, which generates significant heat that, although small compared to ICP, can cause structural deformation depending on the thermal tolerance of the DBD plasma generator components. This structural deformation can impact the RCS measurements, leading to inconsistencies in experimental results and difficulties in accurately determining the plasma’s effects. In fact, in [22], a method was proposed to recognize this thermal effect and compensate for the structural deformation problem. However, it should be noted that it did not provide an analysis of the fundamental approach to reducing the thermal-induced structural deformation.

The aim of this paper is to analyze and address the effects of heat generated by DBD plasma generation systems. A comprehensive system was developed to evaluate the deformation of the DBD plasma generator structure due to the heat generated during plasma generation. Through measurements using a thermal imaging camera, the heat generated by the DBD plasma generator was quantitatively identified. Then, a high-resolution laser was used to measure the degree of heat-induced deformation. Through our study, specific solutions were proposed to mitigate the deformation, and in experiments, the heat was reduced by more than two times, and the deformation was minimized by 43%. Furthermore, a simple method for compensating for the error caused by this minimized deformation is presented.

To demonstrate the validity of the proposed thermal compensation method, RCS measurement experiments were conducted on the structure presented in [18] and a new DBD plasma generator structure. The experimental results show that the accuracy of the simulation and measurement results is improved by using the proposed method.

## 2. Experimental Setup for Measuring Thermal Deformation

### 2.1. Proposed DBD Generator

**Figure 1:**
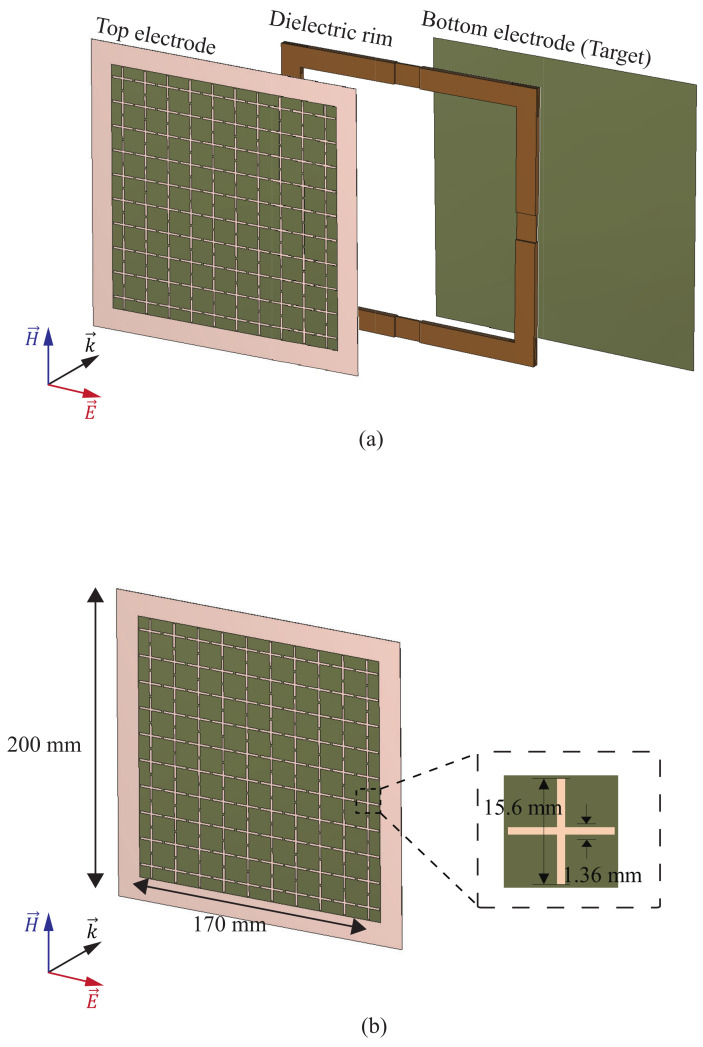
Proposed DBD generator: (**a**) complete structure; (**b**) top electrode.

As plasma is a lossy medium, the RCS can be reduced by attenuating the radar signal as it propagates through. However, the DBD plasma generator used in this paper is a typical generator, a parallel plate capacitor. Therefore, the top electrode that first encounters the radar signal must be configured with a frequency-selective surface (FSS) that allows a specific frequency band signal to pass through it, enabling electromagnetic waves to reach the plasma. The DBD plasma generator structure designed with this principle in mind is shown in Figure 1a.

The top electrode was constructed by arranging cross-dipole cells, designed to pass band in the *X*-band. The bottom electrode, with dimensions of 200 × 200 mm${}^{2}$, also served as the target in this study. To isolate the region between the two electrodes, a dielectric rim with a thickness of 4.572 mm is positioned along the perimeter. The dielectric rim, fabricated using an FR-4 substrate with a permittivity of 4.5, was 15 mm wide. This configuration not only enhances the plasma reduction effect, as mentioned in [18], but also ensures that the pressure between the two electrodes remains independent of the environmental pressure. Consequently, the generator can effectively operate under various pressure conditions.

### 2.2. Experimental Setup for RCS Measurements and Thermal Deformation Analysis of DBD Structures

**Figure 2:**
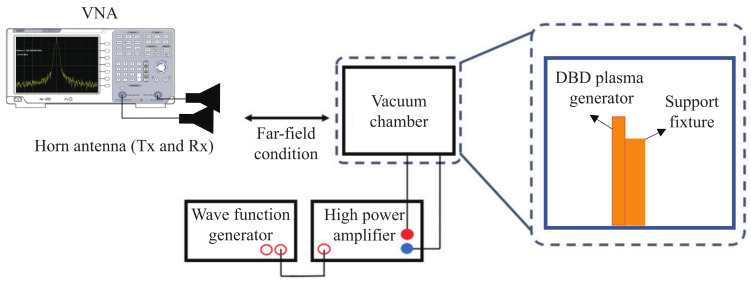
Experimental setup for RCS measurement.

The experimental setup for measuring the RCS is shown in Figure 2. The generator being measured was placed inside an acrylic chamber with a volume of 400 × 300 × 300 mm${}^{3}$. Throughout the experiment, the air pressure in the chamber was maintained at 0.3 atm to match the atmospheric environment. A bias voltage of 10 kV and a driving frequency of 1 kHz were supplied using a 3350 B waveform generator from Keysight and a 10/40 A high-power amplifier from Trek. To satisfy the far-field condition in the frequency range being measured, the distance between the antenna and the target in the acrylic chamber was set to 10 m. A pair of double-ridged horn antennas with a gain of 15 to 18 dBi in the *X*-band and a vector network analyzer (VNA) 37237D from Anritsu with a time-gating function to measure reflections from the generator were used.

(1) $$\sigma_{target}=\sigma_{r}\frac{\Gamma_{t}-\Gamma_{b}}{\Gamma_{r}-\Gamma_{b}}.$$

The RCS measurement process used in this study is shown in Equation (1). In Equation (1), $\sigma_{r}$ is the RCS of the reference target whose RCS is known. In addition, $\Gamma_{t}$ is the measured reflection coefficient of the target, $\Gamma_{r}$ is the measured reflection coefficient of the reference target, and $\Gamma_{b}$ is the reflection coefficient of the background without the target or reference target. The subtraction removes background noise. By comparing the difference in the RCS before and after plasma generation, the degree of RCS reduction by plasma can be obtained.

In addition, an experimental setup was constructed to measure the thermal deformation of the DBD plasma generator. A camera with manual focus was aligned outside the vacuum chamber to analyze the thermal effects of the DBD plasma generator. Several photos were taken from the top and sides of the chamber to capture different perspectives.

**Figure 3:**
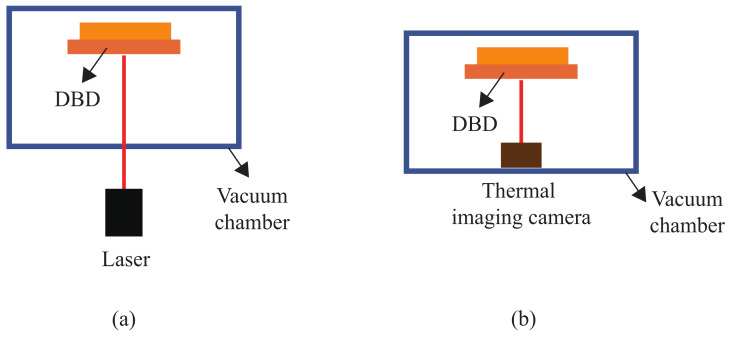
Experimental setup for measuring thermal effects in DBD plasma generators: (**a**) measurement of thermal effects in DBD plasma generators; (**b**) measurement of electrode deformation in DBD plasma generators.

A schematic diagram of the measurement setup to analyze the thermal effects of the DBD plasma generator is shown in Figure 3. As shown in Figure 3a, the OptoNCDT 1420 laser from MicroEpsilon [23] was used to measure the amount of deformation of the top electrode due to heat, and as shown in Figure 3b, FLK-PTI120 from the Fluke thermal imaging camera [24] was used to measure the heat generated by the DBD plasma generator.

### 2.3. Heat Effect in the DBD Plasma System

**Figure 4:**
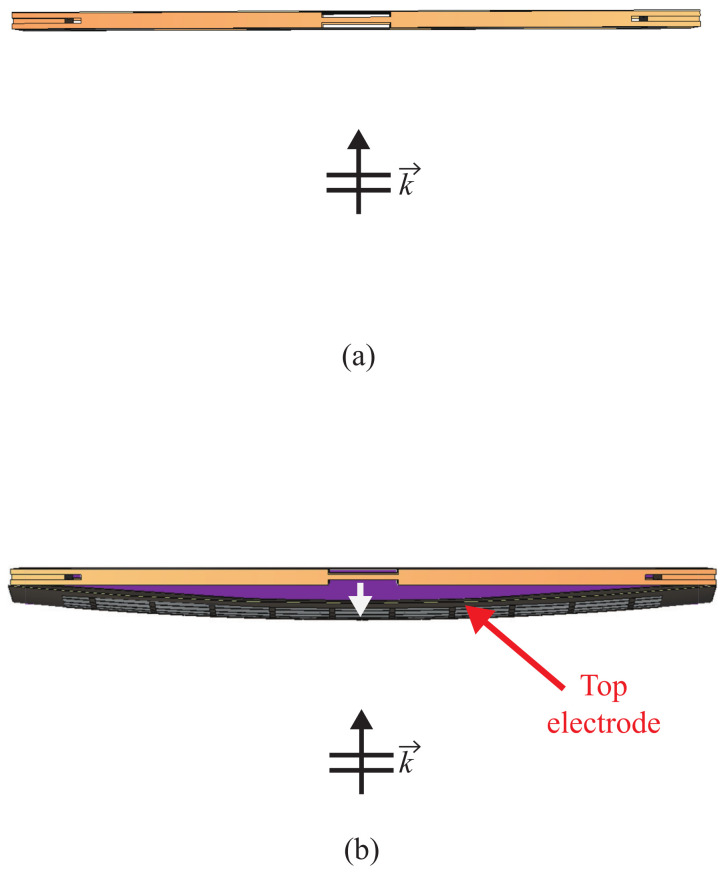
DBD structure deformation by heat (top view): (**a**) plasma off; (**b**) plasma on.

A schematic representation of the deformation of a DBD structure captured using a high-resolution camera when plasma is generated by applying voltage to the DBD generator system is shown in Figure 4. As evident from Figure 4, the application of plasma in the DBD system leads to deformation caused by the generated heat. The figure clearly demonstrates that the top electrode undergoes swelling around the center when the plasma is activated, highlighting the impact of DBD-induced heat. Moreover, the plasma generation process produces high temperatures exceeding 120 °C. Considering that previous studies suggest a maximum temperature of 82 °C or lower for acrylic materials used as DBD supports, it is reasonable to anticipate deformation in the support structure due to heat [17,18]. Therefore, when utilizing a DBD structure that generates significant heat, it becomes essential to employ methods that minimize the heat-induced effects and provide a compensation method.

## 3. Solution for DBD Deformation Due to Heat

**Figure 5:**
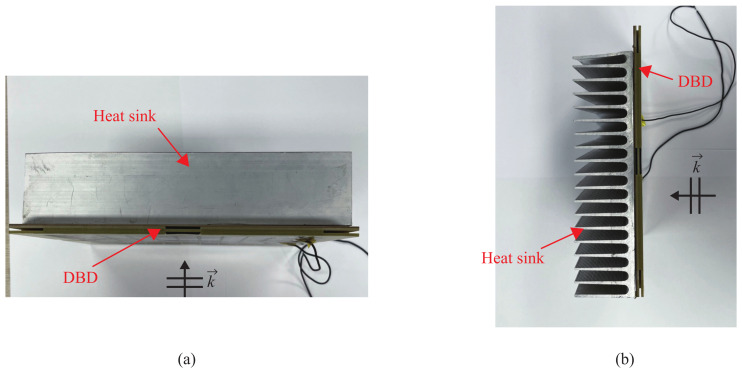
Photographs of the heat sink as the support for the DBD plasma generator: (**a**) top view; (**b**) side view.

To address the issue of heat-induced deformation in the acrylic holder of the conventional DBD plasma generator, the use of an aluminum heat sink is proposed. As depicted in Figure 5, the aluminum heat sink was attached to the back of the bottom substrate using thermal tape. High-temperature-resistant thermal double-sided tape was also employed to integrate the dielectric boundary and the top electrode. In Table 1, the temperature and deformation of the DBD plasma generator are compared with the acrylic support and the aluminum heat sink support when a bias voltage of 10 kV and a driving frequency of 1 kHz were applied, which is higher than the breakdown voltage of 8.0 kV, applied for the stable generation of plasma.

Utilizing the aluminum heat sink support, the heat of the DBD plasma generator was effectively dissipated to the surroundings, resulting in a temperature reduction to 52 °C during the measurements. This temperature is more than twice as low as in the previous setup. Additionally, the expansion of the upper electrode was measured using MicroEpsilon’s laser displacement sensor OptoNCDT 1420. The results showed that the expansion of the upper electrode due to heat was reduced from 2.8 ± 0.3 mm to 1.6 ± 0.3 mm.

With the use of an aluminum heat sink, the deformation of the DBD plasma generator due to heat was dramatically reduced, but it still existed. To reduce the impact of this deformation and achieve more accurate plasma analysis, the following error compensation method was used:

(2) $$\Delta E=D_{a}-D_{b}.$$

Using the previously measured inflation of the top electrode of 1.6 mm, the RCS of the proposed DBD structure with ($D_{a}$) and without ($D_{b}$) inflation is created with a full-wave simulation, respectively. Then, the ΔE can be obtained from the difference between the RCS with ($D_{a}$) and without ($D_{b}$) inflation, respectively. The error ΔE that can be obtained from the difference of the RCS in the two structures is eliminated if no structural deformation occurs. By compensating for these errors with the measured RCS results, thermal deformation is minimized, and a more accurate analysis of plasma effects is possible.

## 4. Results

**Figure 6:**
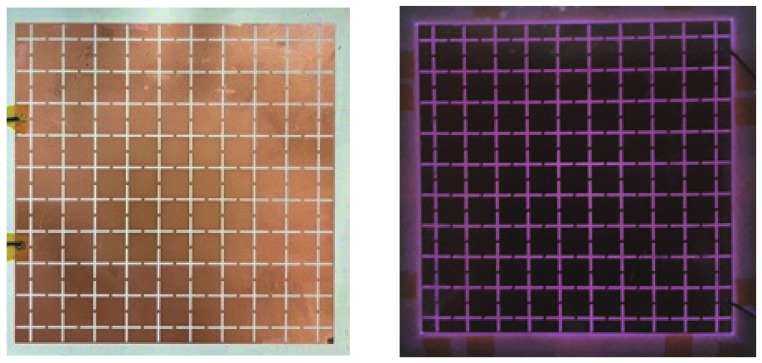
Fabricated type 1 DBD generator: plasma off state (**left**), plasma on state (**right**).

The fabricated DBD generator is shown in Figure 6. To generate plasma, a drive frequency of 1 kHz and a peak-to-peak voltage of 8 kV were applied to the generator, as shown in the left side of Figure 6, and plasma began to be generated inside the generator. Increasing the applied bias voltage further to a peak-to-peak voltage of 10 kV produced stable plasma throughout the generator, as shown in the right side of Figure 6.

**Figure 7:**
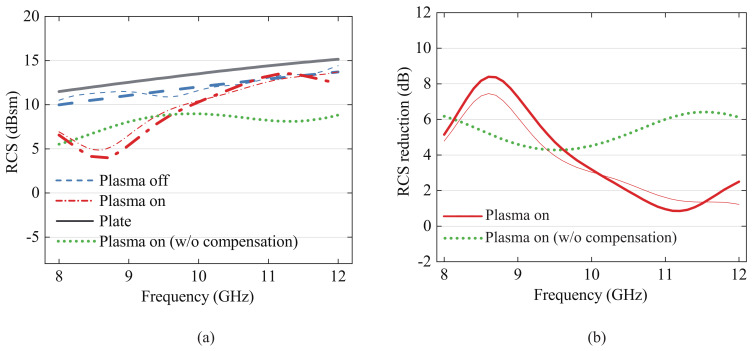
Experimental results (measured (thick) and simulated (thin)): (**a**) comparison of RCS effect of type 1 DBD plasma generator; (**b**) comparison of RCS reduction effect of type 1 DBD plasma generator.

A comparison between the simulation and measurement results of the RCS before and after the proposed error compensation method was implemented is presented in Figure 7. Simulations were performed with commercial software CST [25] using the Drude model, which is a popular model that characterizes the electromagnetic properties of plasma [26,27,28,29]. The full-wave simulation takes into account the deformation of the top electrode, and the plasma angular frequency was simulated to be 83 Grad/s and the collision frequency 684 GHz for 0.3 atm [29]. This corresponded to an electron density of 2.16 × 10${}^{12}$ cm${}^{-3}$. According to the Drude model, this converted to a permittivity of 0.98, with the loss tangent varying between 0.204 and 0.133 in the *X*-band.

The results of the RCS reduction in the plasma on state compared to the plate in the proposed structure are displayed in Figure 7b. The RCS values and the RCS reduction exhibit excellent agreement between the simulated and measured results. Results reveal that by attaching the FSS-based DBD generator to the plate, the RCS is reduced by as much as 1.5 dB. When a bias voltage was applied, and the plasma was generated, the RCS was reduced further by as much as 7.2 dB. The maximum RCS reduction was 8.7 dB at 8.8 GHz.

Furthermore, to verify the proposed error compensation method, in addition to the DBD plasma generator structure in Figure 1, a type 2 DBD plasma generator with a different type of electrode structure was measured by applying the method to minimize the influence of the proposed thermal effect. The top electrode of the type 2 generator is also an FSS based on an alternating array of two crossed dipoles that resonate at different resonant frequencies to increase the bandwidth. The vertical and horizontal lengths of the two crossed dipoles are 16.6 mm and 14.0 mm, respectively. The RCS of the type 2 structure was also measured and analyzed using the method presented in this article.

**Figure 8:**
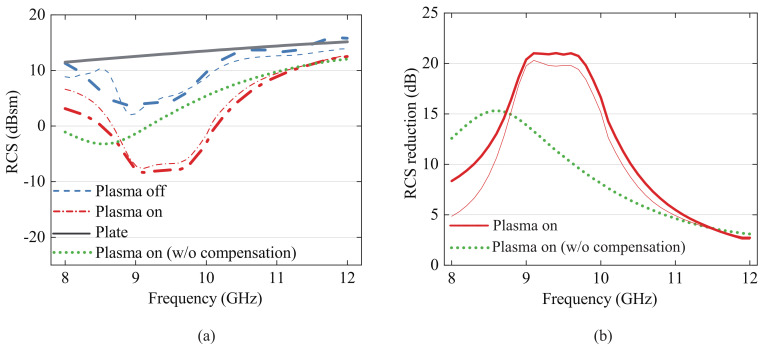
Experimental results (measured (thick) and simulated (thin)): (**a**) comparison of RCS effect of type 2 DBD plasma generator; (**b**) comparison of RCS reduction effect of type 2 DBD plasma generator.

The simulated and measured results of the RCS and RCS reduction considering the implementation of the proposed error compensation method in the type 2 DBD structure are compared in Figure 8a,b. An aluminum heat sink was used to minimize the deformation of the upper electrode to 1.6 mm, and the plasma angular frequency was simulated to be 87 Grad/s and the collision frequency 684 GHz for 0.3 atm [29]. This corresponds to an electron density of 2.38 × 10${}^{12}$ cm${}^{-3}$. According to the Drude model, this converts to a permittivity of 0.98, with the loss tangent varying between 0.214 and 0.122 in the *X*-band.

By applying the heat effect compensation method described in Equation (2), it becomes evident that the simulation and measurement results exhibit exceptional agreement for both DBD structures. This noteworthy alignment validates the effectiveness of the compensation technique and further strengthens the accuracy of the measurements. Moreover, by mitigating the impact of top electrode deformation with this compensation method, the RCS reduction effect can be reliably estimated. This estimation allows for precise analysis of the plasma characteristics and significantly enhances the consistency and reliability of the measurement outcomes.

The newest reported plasma RCS reduction techniques are presented in Table 2. The type 2 DBD plasma generator, utilizing the proposed method, exhibits superiority in maximum RCS reduction and bandwidth characteristics compared to previously reported plasma-based methods, despite operating in a lower frequency range. The method proposed in [30] shows potential effectiveness even in bistatic cases, but the requirement to use low-pressure fluorescent lamps may make it less practical.

In this study, the FSS was designed assuming the absence of the plasma. However, it is anticipated that the effectiveness would be further increased if the FSS is designed with the presence of the plasma. In this case, the electromagnetic properties of the plasma must be characterized accurately. This aspect remains a topic for future research and improvement.

To summarize this work, a method is presented to accurately determine the extent of RCS reduction due to plasma by minimizing thermal deformation and compensating for the effects of the deformation that nevertheless occurs. The validity of the method is verified by the good agreement between the compensated experimental and simulation results. Furthermore, the method can be utilized to design a generator to compensate for the effect of the deformation. However, its effectiveness is uncertain because the RCS of a distended structure is generally higher than that of a flat structure.

## 5. Conclusions

The aim of this paper was to analyze and address the effects of heat generated by DBD plasma generation systems. A comprehensive system for evaluating the deformation of the DBD structure resulting from the heat generated during plasma generation was developed. A solution for mitigating this issue was proposed, and it was demonstrated, through experiments, that the heat can be reduced by more than two times. Furthermore, a straightforward method for compensating for errors caused by the minimized deformation was presented. This compensation technique enables a more accurate analysis of the RCS effect induced by plasma, thus improving the precision of the measurements. To validate the efficacy of the proposed approach, two types of DBD generator structures were fabricated, and RCS measurements were performed using these structures. Remarkably, the results showed excellent agreement when compared to the simulated data for both DBD structures. This strong correlation between the simulation and measurement results provides further evidence of the accuracy and reliability of the proposed method. In conclusion, the proposed method offers a promising and practical solution for obtaining more accurate RCS measurements in various DBD structures that generate significant amounts of heat. By addressing the heat-induced deformation and implementing compensation techniques, we can effectively improve the precision and reliability of RCS measurements. These advancements have the potential to greatly contribute to the field of DBD plasma systems and facilitate more accurate characterization and analysis in a wide range of applications.

## Figures and Tables

**Table 1:** Effect of thermal deformation on DBDs.

	Acrylic Block	Aluminum Heat Sink
Temperature (°C)	120	52
Degree of deformation (mm)	2.8 (±0.3)	1.6 (±0.3)

**Table 2:** Comparison of plasma RCS reduction techniques.

Reference	Frequency Range (GHz)	Maximum RCS Reduction (dB)	10 dB RCS Reduction BW (%)
[17]	14–18	4.1	0
[18]	8–12	8.1	0
[30]	8–12	10.1	2
This work	8–12	21	22

## Data Availability

The data presented in this study are available on request from the corresponding author.
